# Need and disparities in primary care management of patients with diabetes

**DOI:** 10.1186/1472-6823-14-56

**Published:** 2014-07-10

**Authors:** Alessandra Buja, Rosa Gini, Modesta Visca, Gianfranco Damiani, Bruno Federico, Daniele Donato, Paolo Francesconi, Alessandro Marini, Andrea Donatini, Salvatore Brugaletta, Giorgia Bardelle, Vincenzo Baldo, Mariadonata Bellentani

**Affiliations:** 1 Department of Molecular Medicine of the University of Padova, Laboratory of Public Health and Population Studies, Via Loredan, 18, 35100 Padova, Italy; 2 Toscana Region Healthcare Agency, Florence 50100, Italy; 3 Healthcare Organization Department, National Health Agency, Rome 00100, Italy; 4 Catholic University of Sacred Heart, Rome 00100, Italy; 5 University of Cassino and Southern Lazio, Cassino 03043, Italy; 6 Local Health Unit 16 Padua, Veneto Region, Padua 35100, Italy; 7 Zona Territoriale Senigallia, Regione Marche, Senigallia 60019, Italy; 8 Regione Emilia Romagna, Bologna 40100, Italy; 9 ASP 7 Ragusa, Regione Sicilia, Ragusa 97100, Italy

**Keywords:** Health care research, Quality of care, Prevalence, Inequalities

## Abstract

**Background:**

An aging population means that chronic illnesses, such as diabetes, are becoming more prevalent and demands for care are rising. Members of primary care teams should organize and coordinate patient care with a view to improving quality of care and impartial adherence to evidence-based practices for all patients. The aims of the present study were: to ascertain the prevalence of diabetes in an Italian population, stratified by age, gender and citizenship; and to identify the rate of compliance with recommended guidelines for monitoring diabetes, to see whether disparities exist in the quality of diabetes patient management.

**Methods:**

A population-based analysis was performed on a dataset obtained by processing public health administration databases. The presence of diabetes and compliance with standards of care were estimated using appropriate algorithms. A multilevel logistic regression analysis was applied to assess factors affecting compliance with standards of care.

**Results:**

1,948,622 Italians aged 16+ were included in the study. In this population, 105,987 subjects were identified as having diabetes on January 1^st^, 2009. The prevalence of diabetes was 5.43% (95% CI 5.33-5.54) overall, 5.87% (95% CI 5.82-5.92) among males, and 5.05% (95% CI 5.00-5.09) among females. HbA1c levels had been tested in 60.50% of our diabetic subjects, LDL cholesterol levels in 57.50%, and creatinine levels in 63.27%, but only 44.19% of the diabetic individuals had undergone a comprehensive assessment during one year of care. Statistical differences in diabetes care management emerged relating to gender, age, diagnostic latency period, comorbidity and citizenship.

**Conclusions:**

Process management indicators need to be used not only for the overall assessment of health care processes, but also to monitor disparities in the provision of health care.

## Background

An aging population means that chronic illnesses, such as diabetes, are becoming more prevalent and demands for care are rising. Diabetes mellitus (DM) has become one of the most important public health challenges of the 21^st^ century. Over 150 million adults suffer from DM worldwide, and this number is expected to double in the next 25 years [1,2]. In Italy, it is reported that 12% of Italians aged over 56 years have been diagnosed with type 2 diabetes and the proportion of government healthcare expenditure for diabetes was 14.7% in 2011 [3]. The IDF Diabetes Atlas estimated that healthcare expenditure for diabetes accounted for 11.6% of the total healthcare expenditure worldwide in 2010 [4].

Efforts to guarantee good-quality chronic disease management in the primary care setting (taking a proactive population-based approach to address chronic diseases early in their cycle with a view to preventing their progression and containing potential related complications) must begin with the consideration that the vast majority of patients (e.g. more than 90% of diabetic patients in the United States [5]) receive the bulk of their care at primary care practices and are likely to do so for the foreseeable future. With this in mind, members of primary care teams should organize and coordinate patient care with a view to improving quality of care, also with a view to containing the growth of medical expenditure by limiting costly complications and unnecessary procedures [6].

The Diabetes Quality Improvement Project (DQIP) has developed and implemented a comprehensive set of performance measures for assessing diabetes care and its quality improvement that could be widely accepted and widely implemented. Performance measures retrospectively assess the level of care delivered across the whole population with diabetes. These measures were defined on the grounds of several criteria, including credible evidence of a link between these process measures (information on the frequency with which a certain laboratory or clinical test is prescribed) and major clinical outcomes that can be modified by the health care system’s efforts and intervention [7]. These measures are already widely used in surveillance systems for tracking and monitoring the quality of primary care at population level. The clinical challenge that remains therefore concerns how to improve physicians’ impartial adherence to evidence-based practices for all patients, and to monitor any clinically unjustified, systematic differences and deviations from best care practices.

The aims of the present study were: to ascertain the current prevalence of patients treated for diabetes, stratified by age, gender and citizenship; to identify the rate of compliance with recommended guidelines for monitoring diabetes in Italy; and to establish whether disparities exist in the quality of diabetes patient management. Data were obtained from the Italian VALORE project, a national scheme designed with the purpose of assessing quality of care for chronic diseases and the organization of primary health care services [8].

## Methods

### Context

Italy is divided administratively into 20 regions, whose governments have the important role of fulfilling the objectives of the National Health Plan at regional level. They are responsible for planning and organizing health care facilities and related activities through regional health departments. They also coordinate and control the local health units (LHU), each of which is an autonomous National Health System (NHS) body that organizes and plans the services in a given area, providing community health care closer to where people live. The LHUs are organized into districts that coordinate all NHS and publicly-funded facilities and services delivered outside the hospital for a portion of the LHU community.

### Participants and dataset

Data for this study were obtained from a research project performed between November 2010 and April 2011, called the “Valutazione dei nuovi modelli organizzativi della Medicina Generale,” (acronym VALORE) and funded by AGENAS (the Italian national agency for regional healthcare services) to assess certain organizational features of primary care, such as the management of diabetes mellitus and other chronic diseases, as explained in detail elsewhere [8]. Six Italian regions, two in northern Italy (Lombardy and Veneto), three in central Italy (Emilia Romagna, Tuscany and Marche), and one in southern Italy (Sicily) took part in the VALORE project. One or two LHUs in each region were involved in the study (8 in all) and, for each LHU, two to four district administrations (21 in all) shared their data.

The dataset for analysis was collected on all individuals registered with GPs in each area by automatically processing routine LHU administrative databases with the aid of appropriate algorithms.

The cases of diabetes were drawn from this sample using an algorithm prepared by the Tuscany Regional Public Health Agency [9], relying on the diagnoses in hospital discharge records, drug usage as recorded in drug dispensing records, and disease-specific exemptions from health care copayment, as described in detail elsewhere [10]. Compliance with standards of care was assessed on the basis of adherence to three requirements established by the OECD [11] as indicators of the quality of diabetes care at health system level, i.e.

– at least one HbA1c test a year;

– screening for nephropathy at least once a year;

– at least one LDL cholesterol test a year.

Adherence to these requirements was estimated from administrative databases recording drug prescriptions and diagnostic service usage in 2009, as described in detail elsewhere [12]. The reliability of this database was assessed in recently-published papers, which confirmed the consistency of the VALORE database [12] used in the present study with other sources of data, such as primary care medical records and national surveys.

### Statistical analysis

Data were summarized as numbers (percentages) of individuals for categorical variables. After an appropriate descriptive analysis, exact two-sided confidence intervals were constructed based on the binomial proportion.

A multilevel regression model was used to analyze compliance with standards of care.

All patients lost to follow-up during 2009 were excluded from this analysis.

The independent variables had a hierarchical structure with:

– patient level [first-level unit]: gender, age group (16–44, 45–64, 65–74, 75–84, >85 years old), nationality; and clinical covariates: Charlson’s index (no comorbidity, low comorbidity, severe comorbidity), time since diagnosis (dichotomized as ≤ 3 years and > 3 years);

– district of residence level [second-level unit].

We classified nationalities as follows: Italians; immigrants from highly-developed countries (HDC); immigrants from high migratory pressure countries (HMPC) [13]. The HMPC included new Member States of the European Union, Africa, Asia (except for Israel and Japan), and Central and South America; by extension, stateless people were also included in this group. The HDC included the other European countries, North America, Oceania, Israel and Japan.

The level of statistical significance was set a priori at p < 0.01 for all analyses. The analyses were all performed with Stata/SE 12.1.

#### *Ethical issues*

The data analysis was performed on anonymized aggregated data without any chance of individuals being identifiable. The manuscript complies with the Declaration of Helsinki and Italian Law n. 196/2003 on the protection of personal data. The recent Resolution n. 85/2012 of the Guarantor for the protection of personal data confirmed permission to process personal data for medical, biomedical and epidemiological research: details concerning health status can be used as aggregated data for scientific studies [14]. The dataset used in this study is not publicly available. Permission to use non-identifiable individual data extracted from administrative databases for the VALORE project was granted by the ULSS 16 Padova, the ASP 7 Ragusa, the Assessorato Politiche per la Salute Emilia Romagna, the Zona Territoriale Senigallia, the Regione Lombardia, and the Agenzia Regionale di Sanità della Toscana, which are responsible for any use of the data concerning their respective populations. A disclosure statement was also submitted to the ethics committees of the Local Health Units in the areas participating in the study.

Approval for the use of encrypted and aggregated data was also obtained from the Italian College of General Practitioners.

## Results

The number of individuals over 16 years old as at 1 January 2009 registered with a GP in the areas considered amounted to 1,948,622. The case ascertainment algorithm identified 105,987 diabetic patients in this population. Table 1 summarizes the characteristics of these diabetic patients.

**Table 1:** Characteristics of the sample

**Variables**			
*Gender*	Female	48.45%	(51,356)
	Male	51.55%	(54,631)
*Age group*	16-44	5.11%	(5,411)
	45-64	26.96%	(28,574)
	65-74	29.47%	(31,237)
	75-84	27.65%	(29,303)
	> 85	10.81%	(11,462)
*Citizenship*	Italian	96.82%	(96,992)
	Highly-developed country	0.29%	(291)
	High migratory pressure country	2.82%	(2,820)
*Charlson’s index*	No comorbidities	67.41%	(71,451)
	Mild comorbidities	13.45%	(14,250)
	Severe comorbidities	19.14%	(20,286)
*Time since diagnosis*	>3 years	66.19%	(70,155)
	≤ 3 years	33.81%	(35,832)

The prevalence of diabetes in the population aged ≥16 years was 5.43% (95% CI 5.33-5.54) overall, 5.87% (95% CI 5.82-5.92) among males, and 5.05% (95% CI 5.00-5.09) among females. Table 2 shows the prevalence of diabetes by age, gender and citizenship. For Italians over 44 years old, the estimates indicated that women have a significantly lower prevalence of diabetes than men. The table also shows that people from HMPC have a higher prevalence of diabetes than Italians, and the difference reaches statistical significance for adults aged from 45 to 74, in both males and females.

**Table 2:** Prevalence of diabetes by gender, age group and citizenship

**Citizenship**	**Italian**	**HDC**	**HMPC**
	**(n = 1,710,780)**	**(n = 10,935)**	**(n = 110,402)**
**Males**			
*16-44* (n = 415,829)	0.59% (95% CI 0.57-0.62)	0.29% (95% CI 0.06-0.52)	1.01% (95% CI 0.91-1.10)
*45-64* (n = 296,681)	5.84% (95% CI 5.75- 5.93)	2.94% (95% CI 1.96-3.91)	7.13% (95% CI 6.67-7.59)
*65-74 (*n = 118,104)	14.65% (95% CI 14.44-14.86)	14.43% (95% CI 10.48-18.37)	19.00% (95% CI 16.45-21.54)
*75-84 (*n = 76,283)	18.08% (95% CI 17.80-18.37)	16.94% (95% CI 11.50- 22.37)	20.38% (95% CI 15.49-25.28)
*85+* (n = 23,994)	16.47% (95% CI 15.98-16.97)	13.46% (95% CI 4.18-22.74)	15.79% (95% CI 4.20-27.38)
**Females**			
16-44 (n = 408,106)	0.65% (95% CI 0.62- 0.68)	0.40% (95% CI 0.19 -0.62)	0.99% (95% CI 0.90-1.09)
45-64 (n = 308,053)	3.64% (95% CI 3.57- 3.71)	1.81% (95% CI 1.29-2.34)	4.54% (95% CI 4.19-4.89)
65-74 (n = 135,675)	10.26% (95% CI 10.09 -10.43)	7.25% (95% CI 5.31-9.18)	15.79% (95% CI 13.86-17.72)
75-84 (n = 111,872)	14.08% (95% CI 13.87-14.30)	12.06% (95% CI 8.76-15.37)	17.96% (95% CI 14.25-21.67)
85+ (n = 54,025)	14.49% (95% CI 14.18 -14.80)	10.56% (95% CI 5.51-15.62)	18.48% (95% CI 10.55-26.41)

We found that HbA1c tests had been conducted in 60.50% of our diabetic subjects, LDL cholesterol tests in 57.50%, and creatinine tests in 63.27%, but only 44.19% of the diabetic individuals had undergone a comprehensive assessment.

Regression analyses were performed on the 102,207 diabetic patients who had a full year of follow-up from January 1^st^ to December 31^st^ on 2009. Table 3 shows that differences in diabetes management were associated with gender, age, diagnostic latency period, grade of comorbidity and citizenship. In particular, the table shows that adherence to all management indicators for diabetes was slightly better for females than for males, the former having 10% higher odds of undergoing annual renal function tests. The diabetic patients in the retired age group (65–74 year-olds) had more than twice the odds of undergoing the tests considered than the youngest age group (16–44). Subjects diagnosed ≤ 3 years previously were tested more frequently than patients with more longstanding diabetes. Diabetics from HMPC were monitored less than Italian citizens, while there was no such difference between the latter and diabetics coming from HDC.

**Table 3:** Multilevel logistic regression with indicators of adherence to diabetes management guidelines as dependent variables

**Annual HbA1c test**		**OR**	**95% LL**	**95% UL**	***p***
Gender (male = ref.)	Female	1.05	1.02	1.08	*<0.001*
Age group (16-44 = ref.)	45-64	1.73	1.63	1.84	*<0.001*
	65-74	2.14	2.01	2.28	*<0.001*
	75-84	1.78	1.67	1.89	*<0.001*
	85+	0.98	0.91	1.05	*n.s.*
Time since diagnosis	≤ 3 years	1.35	1.31	1.38	*<0.001*
(>3 years = ref)					
Charlson’s index (ref. = no comorbidities)	Low index	1.05	1.00	1.09	*0.030*
	High index	0.89	0.86	0.92	*<0.001*
Citizenship (reference = Italian)	HDC	0.95	0.75	1.21	*n.s.*
	HMPC	0.74	0.68	0.80	*<0.001*
**Annual screening for nephropathy**		**OR**	**95% LL**	**95% UL**	***p***
Gender (male = ref.)	Female	1.10	1.07	1.14	*<0.001*
Age group (16-44 = ref.)	45-64	1.47	1.38	1.57	*<0.001*
	65-74	2.11	1.98	2.25	*<0.001*
	75-84	2.30	2.16	2.45	*<0.001*
	85+	1.60	1.50	1.73	*<0.001*
Time since diagnosis	≤ 3 years	1.19	1.16	1.23	*<0.001*
(>3 years = ref.)					
Charlson’s index	Low index	1.23	1.18	1.28	*<0.001*
(ref. = no comorbidities)					
	High index	1.60	1.54	1.70	*<0.001*
Citizenship (reference = Italian)	HDC	1.01	0.79	1.30	*n.s.*
	HMPC	0.77	0.671	0.83	*<0.001*
**Annual LDL cholesterol test**		**OR**	**95% LL**	**95% UL**	***p***
Gender (male = ref.)	Female	1.04	1.01	1.07	*0.005*
Age group (16-44 = ref.)	45-64	1.71	1.60	1.82	*<0.001*
	65-74	2.11	1.98	2.25	*<0.001*
	75-84	1.69	1.58	1.80	*<0.001*
	85+	0.80	0.74	0.86	*<0.001*
Time since diagnosis	≤ 3 years	1.17	1.13	1.20	*<0.001*
(>3 years = ref.)					
Charlson’s index	Low index	1.01	0.97	1.05	*n.s.*
(ref. = no comorbidities)					
	High index	0.91	0.88	0.94	*<0.001*
Citizenship (reference = Italian)	HDC	0.94	0.74	1.19	*n.s.*
	HMPC	0.65	0.60	0.70	*<0.001*

## Discussion

This population-based Italian study estimated the prevalence of diabetes in the general population. Our prevalence estimates, calculated on subjects aged 16 or more, resemble those reported by the national public health system’s service for the ongoing surveillance of the adult population, developed by the Italian National Health Institute (Istituto Superiore di Sanità, ISS), which reported a prevalence of 4.96% in the population aged 18-69y in 2009 [15], and the Italian Statistics Institute (ISTAT) indicated an overall 4.8% prevalence of diabetes in 2008 [16]. The populations investigated in these various estimates differed in age of enrollment and the methods used to identify cases of diabetes were also quite different: the present study relied on record-linkage administrative data, whereas the other above-mentioned statistics were based on telephone and personal interviews, respectively, indicating self-reported diagnoses of diabetes.

The prevalence of diabetes by age group in our sample was similar to the findings of a study on the prevalence of diabetes in eight European countries [17], and in the US [18]. The prevalence of the disease decreased in older age, probably due to the longer survival of non-diabetic people or to a cohort effect. There was also gender-related difference, with a higher prevalence of the disease in males than in females (especially among the older adults). These results echo previous prevalence estimates conducted in Europe [17], in the USA (for diagnosed and undiagnosed diabetes) [19] and in a number of other countries [20].

Our results also indicated that immigrants from HMPC have a higher prevalence of diabetes than native Italians, as reported in other studies conducted in Israel [20] and in the US National Estimates on Diabetes [21] (this report did not consider immigrant status, but it showed that Blacks and Hispanics have higher rates of diabetes than Whites).

The present study showed that almost 40% of the whole diabetic population in Italy was not monitored annually in terms of HbA1c, LDL or creatinine levels. Only a few cross-sectional studies in Europe provide details on the care process at population level. In a UK population of patients with diabetes, an HbA1c test had been done within the previous year in 93.5% of cases, a serum cholesterol test in 93.1%, and a creatinine test in 93.8% [22,23]. Another study in Israel found that the care delivered by the health services ensured that almost 85% of patients had annual HbA1c and serum cholesterol tests [20]. Our results are very similar to those reported in the USA for Medicare beneficiaries on two quality measures (annual HbA1c testing and lipid profile measurements) [24], and to the findings of a previous survey conducted in the Italian city of Torino [25]. The differences in process performance between different countries may stem from the way in which primary care is organized and motivated, showing better results in countries where ‘pay for performance’ in primary care schemes are in place, as in the UK. After a quarter of the income of family practitioners in the UK was tied to measures of their performance, the Quality and Outcomes Framework showed that previously-identified quality improvement trends for major chronic conditions were maintained or increased, but the effect plateaued as physicians gained the maximum rewards available. For major chronic conditions like diabetes, financial incentives should be seen as part of a broader quality improvement strategy [26]. This philosophy is consistent with the general literature on quality improvement, which suggests that there is no “magic bullet”, but multiple interventions sustained over time can produce major improvements in care [27]. Other issues may also help to explain the differences in process performance between different countries, such as a different reliance on of multidisciplinary primary healthcare teams, which are important in empowering patients and supporting their day-to-day self-management, different methods for training diabetics and their key carers to maintain effective self-management and monitoring. The differences in performance in different countries should encourage international cooperation to facilitate the exchange of good practice for the purpose of controlling diabetes by creating a diabetes forum for action and monitoring [28].

The present study also aimed to identify disparities in the delivery of care to diabetes patients and it emerged that gender, age, comorbidities, citizenship and time since diagnosis were all independent variables explaining differences in the management of this disease.

The role of education and economic status in disparities in diabetes patients management is still being debated in the literature [29]. In the present study, we looked instead at the influence of citizenship, gender and age. Our data show that immigration from a HMPC reduces the likelihood of a patient’s diabetes being monitored, as shown in another study based on the VALORE project [30]. Other studies detected racial and ethnic differences in the quality of diabetes care too [31,32]. It has also already been demonstrated that immigrants make less use of primary care and are less inclined to contact primary care physicians [33], who are the most often involved in the management of diabetes.

Our data indicate that age is a strong factor influencing the management of diabetes care, irrespective of the duration of the disease and any comorbidities. The elderly and younger adults are less likely to be monitored (an abnormal decline in the quality of care for elderly patients has also been reported in some [34,25], but not all [24] other studies). This has nothing to do with the availability of evidence-based care, and probably relates to the propensity of individuals to seek primary care, as demonstrated in a previous study [35]. Younger adults are likely to be busier in occupational terms and child care, and less likely to get in touch with their doctors, while the elderly are probably less able to reach a physician unassisted. Primary care physicians could provide a more proactive service and actively seek such patients out. These considerations identify a challenge for society and health care organizations to find solutions for any “unequal management” issues [36]. A study on the impact of a major pay-for-performance initiative introduced in the UK primary care services in 2004 (which was expected to promote more proactive health care services) suggested that younger patients (<45 years old) with diabetes benefited less in terms of performance quality and outcomes than older patients, i.e. some age group disparities persisted [37].

Gender differences in adherence to process performance measures were small, but emerged consistently for all processes indicators. Other authors have reported a higher proportion of females receiving optimal follow-up than males [20], and one study found gender differences for diabetes patients also in terms of GP appointments and prescribed medication, again in favor of female gender [38]. This better diabetes management in women could be partly explained by the gender-related difference in the outcome of the disease, which is known to carry a lower CVD- and CHD-related mortality risk for men than for women [39] (though the processes measured concern the minimum recommended for all patients).

Patients whose diabetes had been diagnosed less than 3 years earlier were monitored more carefully than patients with a longer history of disease. This applied to all the indicators considered, and to HbA1c testing in particular. This might be because patients with a relatively recent diagnosis are more likely to have difficulty in controlling their disease parameters and consequently need to be monitored more closely. Alternatively, patients with a more longstanding disease might be less concerned about their diabetes and less compliant with their doctor’s recommendations because their clinical condition has become stable, giving the impression that they would need monitoring less frequently. The recommended minimal tests for monitoring diabetes should nonetheless be offered to all patients, irrespective of how long they have been known to have diabetes.

The present study shows that patients with high comorbidity rates were monitored less thoroughly in terms of HbA1c and LDL tests, than those with no comorbidities. Another study [25] also found that a cardiovascular event, for example, distracts the doctor’s and patient’s attention from the need to monitor their diabetes. The onset of other medical issues and the arrival on the scene of other specialists (less sensitive to the diabetes issue) might plausibly diminish the intensity of diabetes screening.

The main drawback of the present study lies in that it cannot separate the effects of socio-demographic determinants on diabetes management, distinguishing between the influence of patients’ socio-demographic features on their different degrees of adherence to the prescribed monitoring practices on the one hand, and differences in physicians’ monitoring practices depending on their patients’ socio-demographic features on the other. Although different strategies would be needed to tackle these two possible causes of shortcomings in health care provision, the message that comes from the present study – that efforts are needed to overcome inequalities in diabetes patient management - is important, whatever the causal factors involved and their relative contributions.

Our study has other limitations. First of all, not all socio-economics factors were considered (patients’ formal education was not known, for instance). Secondly, only process measures were assessed in the present study, but whether or not closer monitoring is necessarily linked to better intermediate or final outcomes for diabetes patients is still being debated [40]. One recent study [41] found that patients receiving the lowest-quality care, as measured in terms of the fulfillment of quality-of-care indicators based on screening guidelines similar to those considered here, carried a higher risk of all-cause mortality and cardiovascular morbidity than those receiving the highest-quality care. Another possible shortcoming of our study lies in that using record linkage may have failed to identify some diabetic patients. The validity of our record linkage procedures, based on three administrative sources (records of hospital discharges, drug prescriptions and payment exemptions for chronic diseases) was addressed in two recent studies [10,42]. Both reported that these routinely-collected data enable disease prevalence to be estimated just as accurately as in other studies conducted in Italy using more costly and time-consuming methods. Another recent study [43] showed that using an additional source (the Diabetes Service’s integrated database) led to the identification of a higher proportion of diabetics, particularly among the elderly. This could be because patients whose condition is treated with lifestyle management alone will not be identifiable from the administrative databases unless they have applied for a disease-specific exemption from healthcare co-payment [11]. Elderly diabetics living in rest homes would also go undetected because rest homes obtain and distribute any drugs needed by residents without using prescriptions for named patients. Measures of compliance with standards of care have shown, however, that these rest home residents receive much the same care as the population identified from clinical data collected by general practitioners [12].

The strength of our study lies in that it was conducted using an unrestricted and unselected population of primary care patients with diabetes mellitus, enabling us to estimate the prevalence of the disease and primary care performance measures.

## Conclusion

In conclusion, management indicators need to be used not only for the overall assessment of a process but also to check for disparities in the provision of health care. Based on such indicators, the present study pointed to numerous opportunities for improving diabetes management, particularly in younger people, patients with longstanding disease, and immigrants. Diabetes is growing significantly and rapidly, so all efforts to take early action and ensure the impartial management of this disease will have a major impact on the long-term health and economic costs for the diabetic population.

## Competing interests

The authors have no financial or other competing interests to disclose.

## Authors’ contributions

Authorship: guarantors for the integrity of the study: AB and RG; study conception and design: MV, MB, GD, BF, DD; intellectual content: GD, BF, PF, AM, AD, SB, MB; statistical analysis: AB, RG; data interpretation: AB; manuscript writing: GB, VB, AB. All authors have read and approved the final manuscript.

## Pre-publication history

The pre-publication history for this paper can be accessed here:

http://www.biomedcentral.com/1472-6823/14/56/prepub
